# Bioaccumulation and detoxification of trivalent arsenic by *Achromobacter xylosoxidans* BHW-15 and electrochemical detection of its transformation efficiency

**DOI:** 10.1038/s41598-021-00745-1

**Published:** 2021-10-29

**Authors:** Farzana Diba, Md. Zaved Hossain Khan, Salman Zahir Uddin, Arif Istiaq, Md. Sadikur Rahman Shuvo, A. S. M. Rubayet Ul Alam, M. Anwar Hossain, Munawar Sultana

**Affiliations:** 1 Department of Microbiology, University of Dhaka, Dhaka, 1000 Bangladesh; 2 Institute of Tissue Banking and Biomaterial Research (ITBBR), Atomic Energy Research Establishment (AERE), Savar, Dhaka, 1349 Bangladesh; 3 Department of Chemical Engineering, Jashore University of Science and Technology, Jashore, 7408 Bangladesh; 4 Department of Stem Cell Biology, Faculty of Arts and Sciences, Kyushu University, Fukuoka, Japan; 5 Graduate School of Medical Sciences, Kumamoto University, Kumamoto, Japan; 6 Department of Microbiology, Noakhali Science and Technology University, Noakhali, Bangladesh; 7 Department of Microbiology, Jashore University of Science and Technology, Jashore, 7408 Bangladesh; 8 Present Address: Jashore University of Science and Technology, Jashore, 7408 Bangladesh

**Keywords:** Biotechnology, Microbiology, Ecology, Environmental sciences

## Abstract

Arsenotrophic bacteria play an essential role in lowering arsenic contamination by converting toxic arsenite [As (III)] to less toxic and less bio-accumulative arsenate [As (V)]. The current study focused on the qualitative and electrocatalytic detection of the arsenite oxidation potential of an arsenite-oxidizing bacteria *A. xylosoxidans* BHW-15 (retrieved from As-contaminated tube well water), which could significantly contribute to arsenic detoxification, accumulation, and immobilization while also providing a scientific foundation for future electrochemical sensor development. The minimum inhibitory concentration (MIC) value for the bacteria was 15 mM As (III). Scanning Electron Microscopy (SEM) investigation validated its intracellular As uptake capacity and demonstrated a substantial association with the MIC value. During the stationary phase, the strain’s As (III) transformation efficiency was 0.0224 mM/h. Molecular analysis by real-time qPCR showed arsenite oxidase (*aio*A) gene expression increased 1.6-fold in the presence of As (III) compared to the untreated cells. The immobilized whole-cell also showed As (III) conversion up to 18 days. To analyze the electrochemical oxidation in water, we developed a modified GCE/P-Arg/ErGO-AuNPs electrode, which successfully sensed and quantified conversion of As (III) into As (V) by accepting electrons; implying a functional As oxidase enzyme activity in the cells. To the best of our knowledge, this is the first report on the electrochemical observation of the As-transformation mechanism with *Achromobacter* *sp*. Furthermore, the current work highlighted that our isolate might be employed as a promising candidate for arsenic bioremediation, and information acquired from this study may be helpful to open a new window for the development of a cost-effective, eco-friendly biosensor for arsenic species detection in the future.

## Introduction

Arsenic (As) is a hazardous compound that is listed among the World Health Organization’s ten major chemicals of public health concern. It is usually found in the earth’s surface, groundwater, sediment soil, and air^1^. The major reasons behind the entrance of As in the ecosphere are natural and human activities^2^. Excessive pumping of groundwater can increase As levels in irrigation and drinking water in Southeast Asia. Iron-reducing bacteria utilize hydrous ferric oxide through reductive dissolution mechanism which is another most important cause of the elevated level of As in groundwater in Bangladesh^3^. WHO and Environmental Protection Agency (EPA) recommended 10 μg/L arsenic as an admissible limit for drinking water^4^. In Bangladesh, the standard limit of arsenic in drinking water is 50 μg/L^5,6^.

Globally, about 170 million people are affected through the consumption of arsenic-contaminated drinking water containing > 10 µg/L arsenic^7^. It has been estimated that about 110 million Asian people use arsenic-rich water for consumption and household purpose^8^. Seventy-five million Bangladeshis (nearly half the population) of 59 (out of 64) districts are exposed to arsenic levels greater than 50 µg/L contaminated water^9^. Among them, the most affected districts are Chandpur (90%), Munshiganj (83%), Gopalganj (79%), Madaripur (69%), Noakhali (69%), Sathkhira (67%), Cumilla (65%), Faridpur (65%), Shariatpur (65%), Meherpur (60%), Bagerhat (60%)^10^. Department of Health Engineering (DPHE) surveyed and found that 29% of tube well out of the 4.95 million were arsenic-contaminated^11^.

International Agency for Research on Cancer (IARC) classified arsenic as category 1 human carcinogen^12^ that is about four times more poisonous than mercury^3^. Long-term consumption of As is the major reason for arsenicosis with symptoms such as diarrhea, vomiting, blood in the urine, hair loss, and more convulsion. Skin infection, abdominal pain, lung, kidney, and bladder problems have also been reported in arsenicosis patients. According to the report by the Directorate General of Health Services of Bangladesh (DGHS), about 65,910 arsenicosis cases had been reported from arsenic-contaminated areas of Bangladesh since 2012^5^. About 21.4% of all deaths occurred in the highly affected area of Bangladesh containing As above WHO permissible limit^13^.

As (III) is more predominant, soluble, and 100 times more poisonous^14^ than As(V) in the anoxic environment^15,16^ as well as difficult to mitigate, whereas As (V) is removed more effectively than As (III) by a conventional method such as precipitation and adsorption^17^. However, traditional procedures are costly and harmful to the environment. Bacteria-mediated arsenic resistance has a crucial contribution in As geocycle^18^. They use harmful arsenic as an energy source for metabolic activity and survival via biosorption, intracellular bioaccumulation, and enzymatic conversion to a less toxic oxidation state^19^. Diverse taxonomic classes of arsenic metabolizing microorganisms such as *Lactobacillus* *sp*., *Stenotrophomonas*, *Alcaligenes*, *Rhodococcus Bacillus* *sp*., *Kocuria* *sp*., *Herminiimonas* *sp*., *Lysinibacillus* *sp*., *Pseudomonas* *sp*., *Aliihoeflea* *sp*., *Achromobacter xylosoxidans*, etc. have already been reported to resist high As concentrations and can contribute in bioremediation of As from groundwater^20–26^. Many bacterial species were found to be involved in both heterotrophic and autotrophic As (III) oxidation^6,27–29^. The detoxification process of As (III) by these bacteria is catalyzed by protoplasmic arsenite oxidase enzymes (AIO and ARX) from the dimethyl sulfoxide (DMSO) reductase family^27,30–39^. Biological oxidation procedures utilizing indigenous arsenite oxidizing bacteria having bioaccumulation capacity could be an alternative for arsenic biomining.

In previous studies, arsenotrophic bacteria are usually retrieved from highly arsenic-contaminated areas such as mines (Coal, gold, etc.), tannery and industrial effluent, agricultural soil, wastewater, hot spring. Our strain *A. xylosoxidans* BHW-15 was reported as a novel heterotrophic arsenite oxidizing bacteria^26^ with a high MIC value isolated from the tube well water. To date, it showed the highest As transformation and accumulation ability compared to the other reported arsenite oxidizing strains in Bangladesh. Furthermore, compared to other arsenic metabolizing bacteria, *A. xylosoxidans* BHW-15 possess a distinctive enrichment of metal resistance genes islands that reflects its high As transformation capacity. Considering its ease of availability, efficiency, and genetic integrity, we chose this strain as a candidate for our bioremediation investigation.

For As bioremediation, an important concern is the detection of arsenic species. In Bangladesh, we usually use colorimetry, atomic absorption spectroscopy (AAS), inductively coupled plasma mass spectroscopy (ICP-MS) for As speciation. Nowadays, electrochemical sensor-based techniques using microorganisms are more advantageous over traditional techniques such as in terms of simplicity, cost-effectivity, portability, high sensitivity, ease of analysis^40–42^. The contribution of the **ɤ**-Proteobacteria consortium in the anaerobic transformation of As (III) in groundwater has already been reported where the oxidation was stimulated by a polarized graphite electrode^43^.

Bangladesh and many underdeveloped countries are still ahead of such research due to a lack of expertise and limited knowledge in arsenic microbial ecology research. Therefore, in this study we investigated the qualitative and electrochemical detection of toxic arsenic biotransformation by *Achromobacter xylosoxidans* BHW-15 and its molecular basis by arsenite oxidizing gene expression analysis, demonstrating its role in arsenic detoxification. To date, we have reported the first electrochemical observation of the As-transformation process by *Achromobacter sp.* using a modified GCE/P-Arg/ErGO-AuNPs electrode, which will open the way for future biosensor development research.

## Results

### Determination of minimal inhibitory concentration (MIC)

MIC at different arsenite concentrations was determined to understand the ability of the strain *A. xylosoxidans* BHW-15 to withstand a higher concentration of As. The isolate showed the ability to grow at high concentrations up to 15 mM As (III).

### Scanning electron microscopy (SEM) analysis

**Figure 1:**
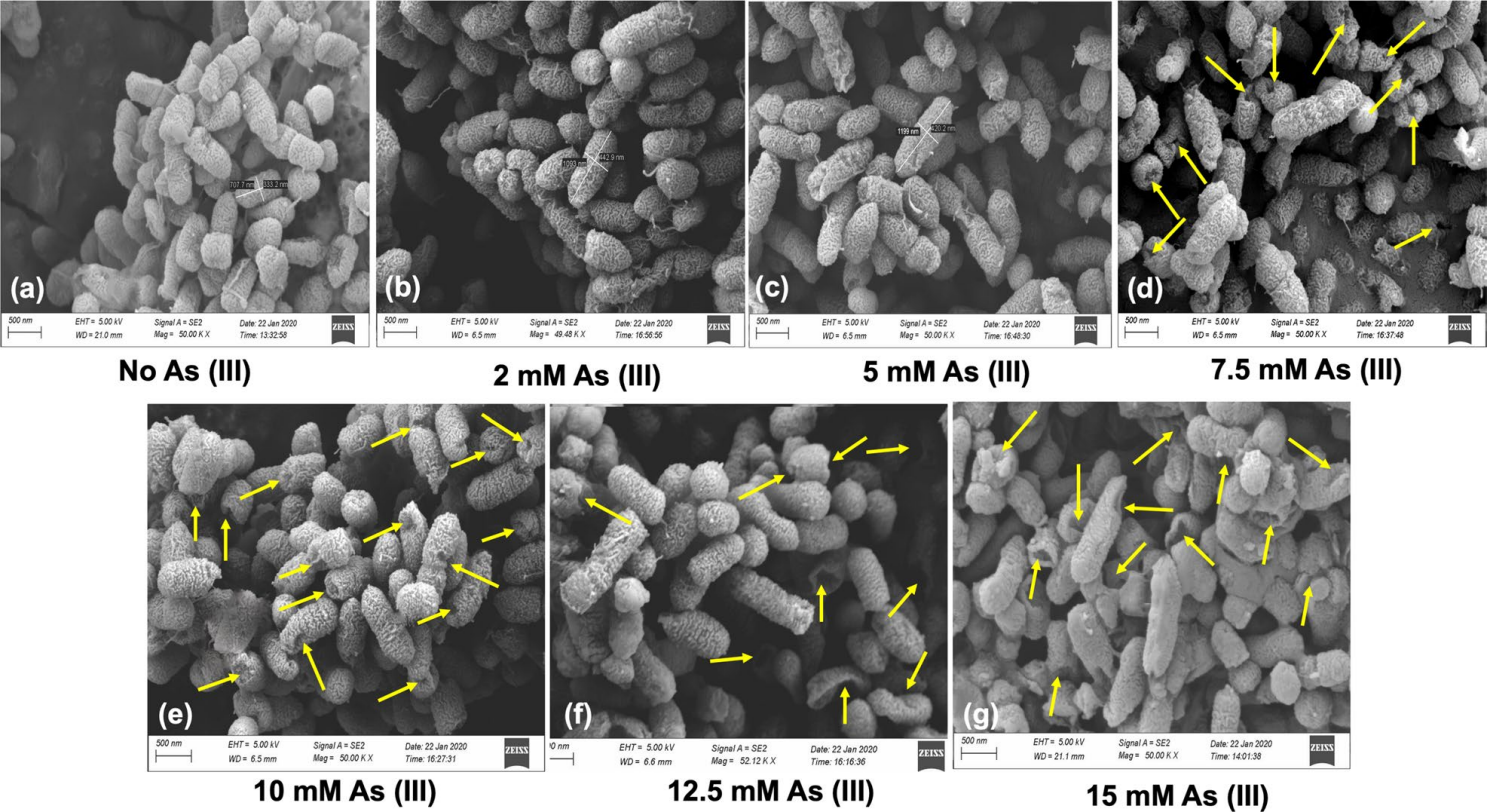
SEM micrographs of *Achromobacter xylosoxidans* BHW15. (**a**) Untreated BHW15 cells. The cell surface volume of BHW15 increased after treatment with 2 mM (**b**) and 5 mM As (III) (**c**). After treatment with 7.5 mM As (III), small holes were appearing in the cells (**d**). Some dimples were found during exposure with 10 mM As (III) (**e**). While treated with 12.5 mM As (III), multiple cavities and few damaged cells were observed (**f**). Lysed cells and debris were seen during treatment with 15 mM arsenite (**g**) (*Yellow arrows indicate the changes inside the cells).

Scanning electron microscopy (SEM) was used for the observation of in-vitro As bioaccumulation by the strain. SEM micrographs showed distinct changes in cell size of the arsenic-treated bacteria at different arsenite concentrations compared to untreated bacteria where the volume of the treated cell increased, 1.5-fold increase at 5 mM arsenite concentration than the untreated cells, which were found shorter (707.7 nm) in length (Fig. 1a,b). However, the cell surface of the untreated cells and cells treated with 2 mM and 5 mM arsenite was intact. A simultaneous increase in cell volume for the treated cell was observed at 2 mM and 5 mM arsenite without any damage, indicating the presence of adaptation and survival mechanism in the treated cells to accumulate arsenite from the media for their metabolic activity while also protecting themselves from the toxic effect of arsenite at high concentrations (Fig. 1b,C).

At 7.5 mM As (III) concentration, a single hole of various shapes was detected on their surface typically towards the septal areas (Fig. 1d). After exposure to 10 mM As (III), there were some dimples in bacterial cells. However, the bacteria were undamaged, shortened, and more compact (Fig. 1e). The bacterial cells were shorter when exposed to 12.5 mM arsenite. Although most of the cells were unharmed, the toxic effect of arsenite caused many cavities and only a few cells were affected (Fig. 1f). The MIC value of *A. xylosoxidans* BHW-15 was 15 mM; at this concentration, we found many lysed cells and cell debris due to burst with a deep dent in their cell wall (Fig. 1g).

### Whole-cell immobilization of *A. xylosoxidans* BHW-15

**Figure 2:**
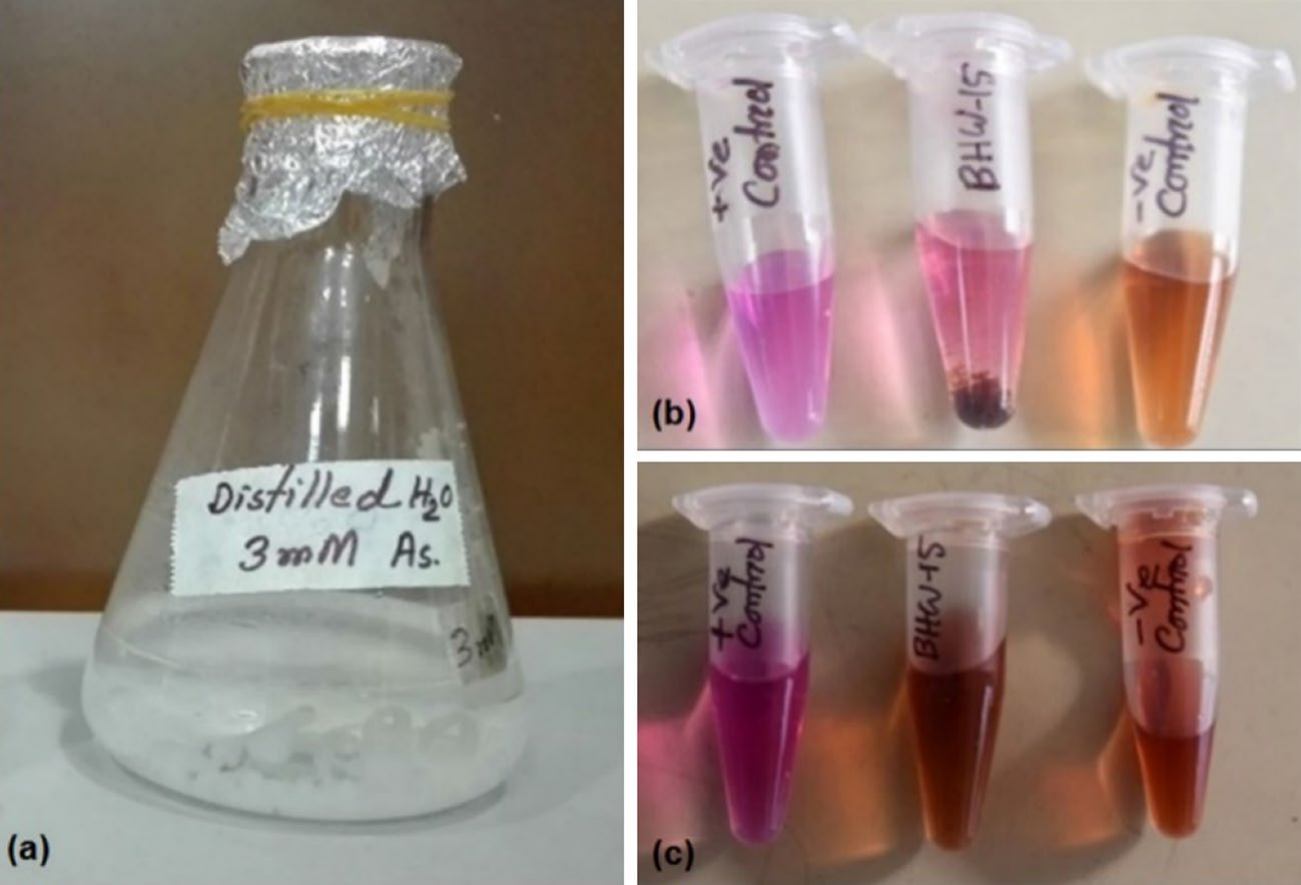
Immobilization of BHW15 in calcium alginate beads and incubation in arsenite containing water (**a**); phenotypic detection after 18 days (**b**); phenotypic detection after 24 days (**c**).

We evaluated the whole-cell immobilization ability of *Achromobacter xylosoxidans* BHW-15 to choose it as a suitable candidate for As detoxification. This isolate was immobilized in calcium alginate beads and the beads were incubated at 30°C with deionized water supplemented with 3 mM sodium arsenite (Fig. 2a). Every 24 h, the conversion was phenotypically assessed by potassium permanganate. The observation is illustrated in Table 1. Potassium permanganate is an oxidizing agent which has a characteristic pink color. It can oxidize arsenite to arsenate. Potassium permanganate loses its pink color when it oxidizes arsenite. On the contrary, in arsenate solution, potassium permanganate retains its pink color as there remains nothing to oxidize indicating conversion of arsenite to arsenate by bacteria. KMnO_4_ pink color retention was observed on the 4th day after incubation of immobilized bacteria in arsenite solution and the intensity of pink color formed after KMnO_4_ addition increased for the next consecutive two days. On the 7th day, the beads were transferred first time to fresh 3 mM sodium arsenite solution. On the 10th day, pink color retention of KMnO_4_ was observed in the new sodium arsenite solution. As the pink color intensity increased in the following 2 days, the beads were second time transferred to fresh 3 mM sodium arsenite solution for the 13th day. In this case, a similar pattern of arsenite oxidation detection was observed. On the 16th day of immobilization, pink color retention of KMnO_4_ was recorded for the second changed solution and arsenite conversion continued up to the 18th day (Fig. 2b). The beads were transferred to the fresh sodium arsenite solution third time on the 19th day. The observation continued till 24th day but no arsenite oxidation was detected phenotypically (Fig. 2c). So, immobilized *Achromobacter xylosoxidans* BHW-15 converted arsenite to arsenate for up to 18 days. Negative control always showed discoloration of KMnO_4_. 3 mM sodium arsenate solution was used as positive control which showed pink coloration after the addition of KMnO_4._

**Table 1:** Day-wise test results of phenotypic detection of arsenite oxidation by KMnO_4_ for immobilized *A. xylosoxidans* BHW-15 isolate.

Medium	Days since immobilization	Pink color formation intensity	Conversion (yes/no)
Initial NaAsO_2_ solution	0	**−**	No
	4	**+**	Yes
	5	**++**	Yes
	6	**+++**	Yes
NaAsO_2_ solution changed first time	7	**−**	No
	10	**+**	Yes
	11	**++**	Yes
	12	**+++**	Yes
NaAsO_2_ solution changed second time	13	**−**	No
	16	**+**	Yes
	17	**++**	Yes
	18	**+++**	Yes
NaAsO_2_ solution changed third time	19	**−**	No
	21	**−**	No
	22	**−**	No
	23	**−**	No
	24	**−**	No

Here, −, +, ++, +++ indicate no color formation, low intensity, moderate intensity, strong intensity respectively.

### Quantitative determination of arsenite oxidation potential

**Figure 3:**
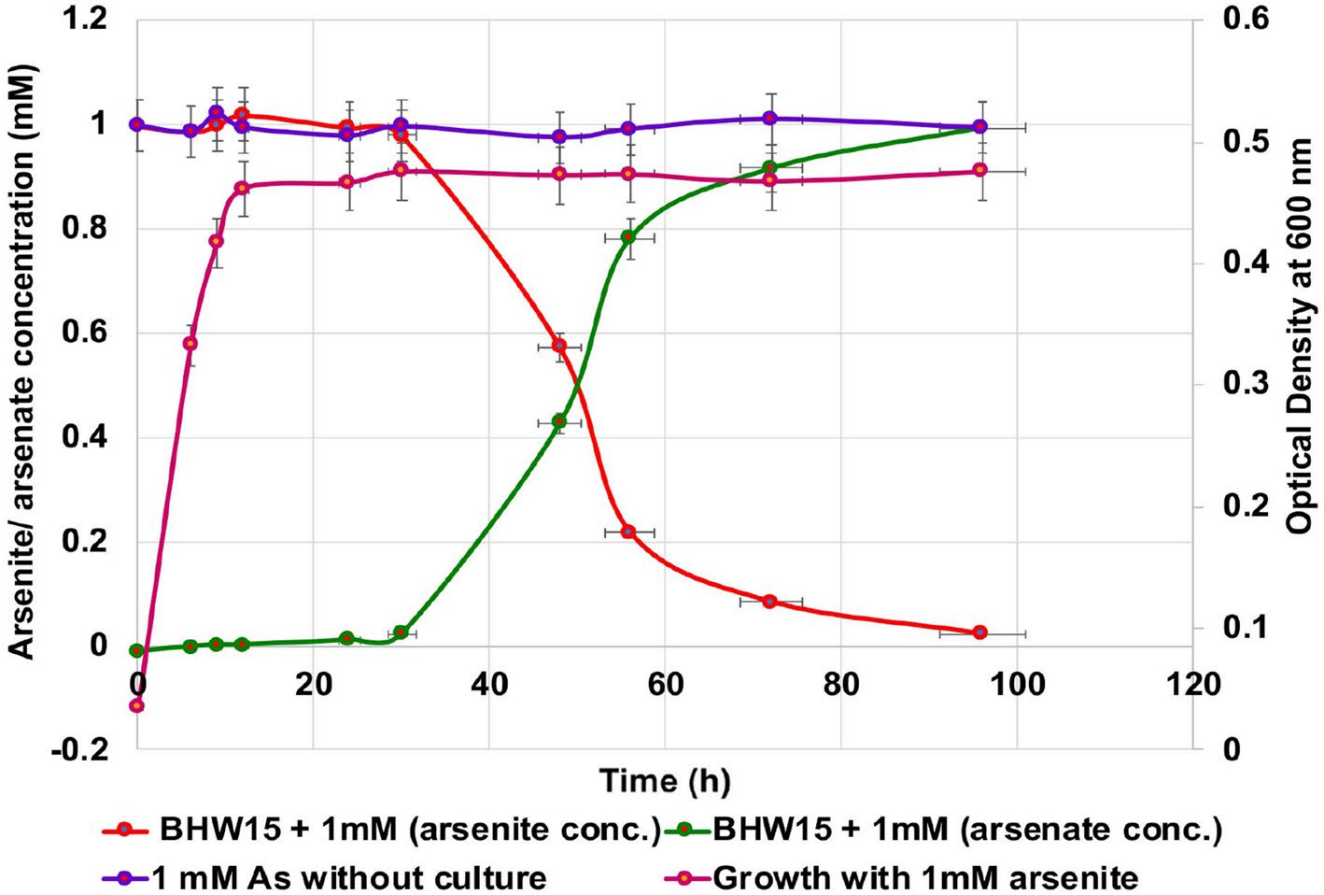
Growth curve and corresponding oxidation of As (III) to As (V) by *Achromobacter xylosoxidans* BHW-15. *A. xlylosoxidans* BHW-15 initiated arsenite conversion at late log phase and efficiently during stationary phase and it converted 1 mM of arsenite at 0.0224 mM/h rate. The error bar represents the deviation of the optical density (OD) measurement data of repeated experiments.

The quantitative arsenite oxidation in a heterotrophic growth medium was investigated as well as the growth curve analysis. Aerobic arsenite oxidation was observed for this isolate. The efficiency was measured concerning the log phase of the growth curve. By the molybdenum blue method, the concentrations of arsenite and arsenate were measured during the phases of growth. Un-inoculated heterotrophic media was used as control media. After 12 h, the growth entered into the stationary phase. Generation time for the isolate was calculated as 4 h and 15 min in presence of arsenite. The isolate was then analyzed to check the arsenic conversion potential. The isolate started converting arsenite efficiently at 30 h after inoculation and finally As (III) to As (V) conversion rate was 0.0224 mM arsenite per hour during their stationary phase (Fig. 3). This experiment was performed twice to confirm reproducibility.

### Analysis of arsenite oxidase (*aio*A) gene expression

**Figure 4:**
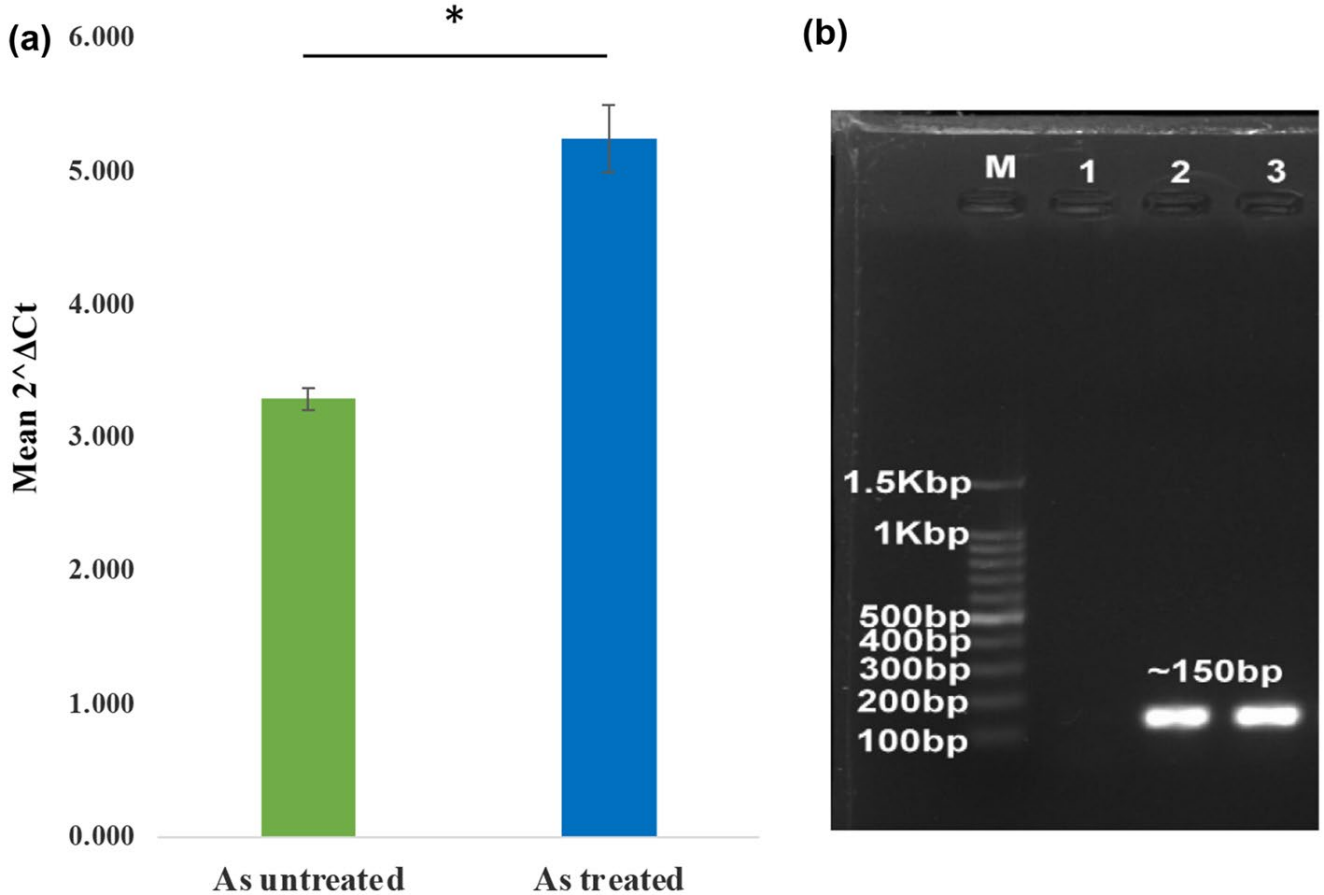
Gene expression analysis of arsenite oxidizing (*aio*A) gene by RT-PCR: (**a**) 2^ΔCt^ plot of As untreated vs As treated sample. Statistical significance (P value) was calculated by t-test P ≤ 0.05*, P ≤ 0.01**, P ≤ 0.001*** [asterisk (*) sign indicated the significance of the test]; (**b**) cDNA samples produced of designed RT-*aio*A primer set. Arsenic (As) untreated and treated *A. xylosoxidans* BHW-15 were screened for RT-*aio*A gene amplicon (~ 150 bp) by conventional PCR using designed RT-primer and observed on 1% agarose gel. Here, M: Marker shown in base pairs; 1: Negative control; 2: As untreated BHW-15; 3: As treated BHW-15. The amplicon size of ~ 150 bp was generated in both samples (Bottom panel). The full-length gel was presented in Supplementary Fig. S3.

The expression pattern of arsenite transforming gene *aio*A (arsenite oxidase) was analyzed in the presence or absence of arsenic. Amplicon size of around 150 bp with similar band intensity was detected for both condition’s cDNA samples (arsenic untreated and treated) by conventional PCR using our designed RT-*aio*A gene primer pairs (Fig. 4b). The full-length gel analysis was displayed in Supplementary Fig. S3.

The gene expression of arsenite oxidase (*aio*A) was compared in As-treated and untreated samples. After the completion of the RT-qPCR, the C_t_ values for both conditions were analyzed. For this purpose, the ΔC_t_ method was used to compare gene expression folds. The expression ratios were normalized based on the control sample after the C_t_ values  were calculated and the relative expression folds for the *aio*A gene at both conditions are shown in Table 2 and Fig. 4a. The expression of the *aio*A gene was 1.6-fold higher in arsenite-treated samples compared to the untreated sample. The amplification plots for both conditions, along with their relative C_t_ values, were displayed in Supplementary Fig. S4a,b. Negative control had not been amplified in any of the cases.

**Table 2:** Arsenite oxidase gene (*aio*A) expression ratios in presence of arsenite comparing with the control condition. Expression of the *aio*A gene was increased in presence of arsenite compared to untreated cells.

Samples	Average C_T_ values at log phase	Average C_T_ values at stationary phase	ΔC_T_ values	*aioA* gene expression folds (2^ΔCT^)	Expression ratio compared to control condition
As untreated (only media)	24.704	22.986	1.718	3.289	3.289/3.289 = 1
As treated	26.601	24.211	2.39	5.242	5.242/3.289 = 1.6

### Electrochemical determination of arsenic transformation

**Figure 5:**
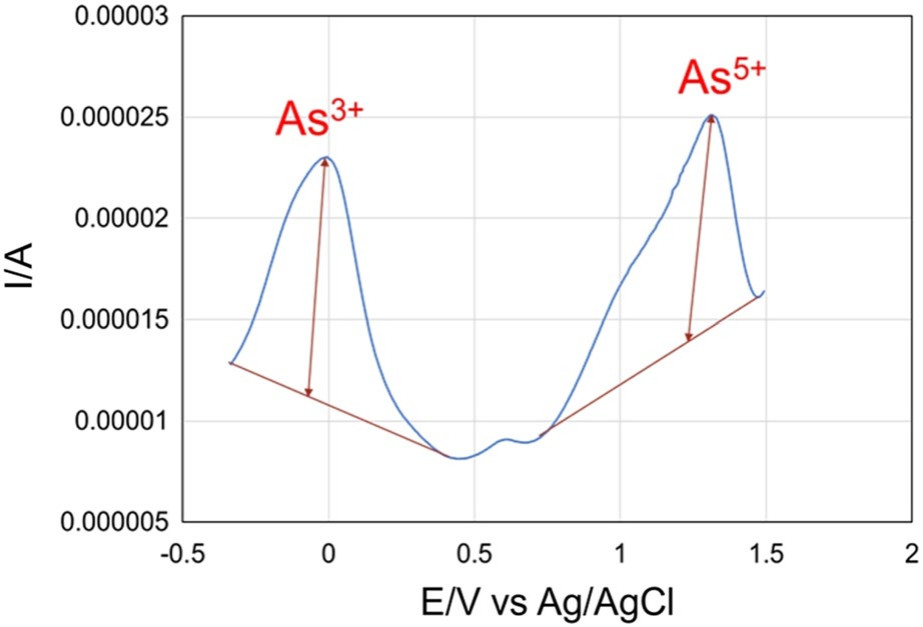
DPVs of 10 ppm arsenic concentration at GCE/P-Arg/ErGO-AuNPs sensor in pH 6.0 phosphate buffer at a scan rate of 50 mV s^−1^. The condition of DPV measurements was initial potential − 1.0 V, end potential 2.0 V, pulse width 50 ms, modulation 50 mV, and pulse period 0.1 s.

Differential pulse voltammetric (DPV) behavior of GCE/P-Arg/ErGO-AuNPs modified electrode was studied in detail for the detection of As (III) and As (V) ions. The modified electrode proposed here showed two oxidation peaks at − 0.02 V and 1.35 V vs Ag/AgCl. These peaks indicate a minor shift towards the negative potential that corresponds to As^0^ → As^3+^ and As^3+^ → As^5+^. Figure 5 represents two peaks where the oxidation of the outer layer As^0^ → As^3+^ corresponds to the first peak at − 0.02 V and the other oxidation peaks at 1.35 V correspond to the electro-oxidation of As^3+^ → As^5+^.

The concentration dependence of arsenic over the GCE/P-Arg/ErGO-AuNPs electrode was evaluated by increasing the arsenic concentration in 0.1 M PBS (pH 6). A linear dependence was observed with different arsenic concentrations for As^3+^ and As^5+^ ions. Due to the release of a greater number of hydrogen and protons, a positive shift in potential was observed for As^5+^ ions. It can be concluded that the proposed electrochemical sensing method is helpful for arsenic speciation with concentration level.

**Figure 6:**
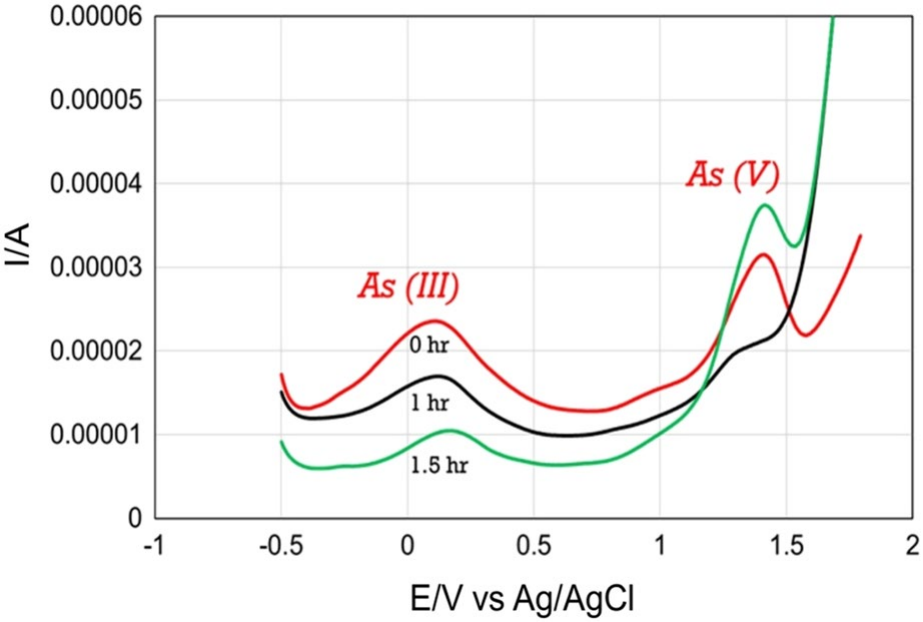
DPV measurements after treatment with *A*. *xylosoxidans* BHW-15. DPVs were obtained after treatment of arsenic solution with *A*. *xylosoxidans* BHW-15 for 0 h, 1 h, and 1.5 h respectively. Electrochemical measurement was done with a modified GCE/P-Arg/ErGO-AuNPs sensor in pH 6.0 phosphate buffer at a scan rate of 50 mV s^−1^. The condition of DPV measurements was initial potential − 1.0 V, end potential 2.0 V, pulse width 50 ms, modulation 50 mV, and pulse period 0.1 s.

**Figure 7:**
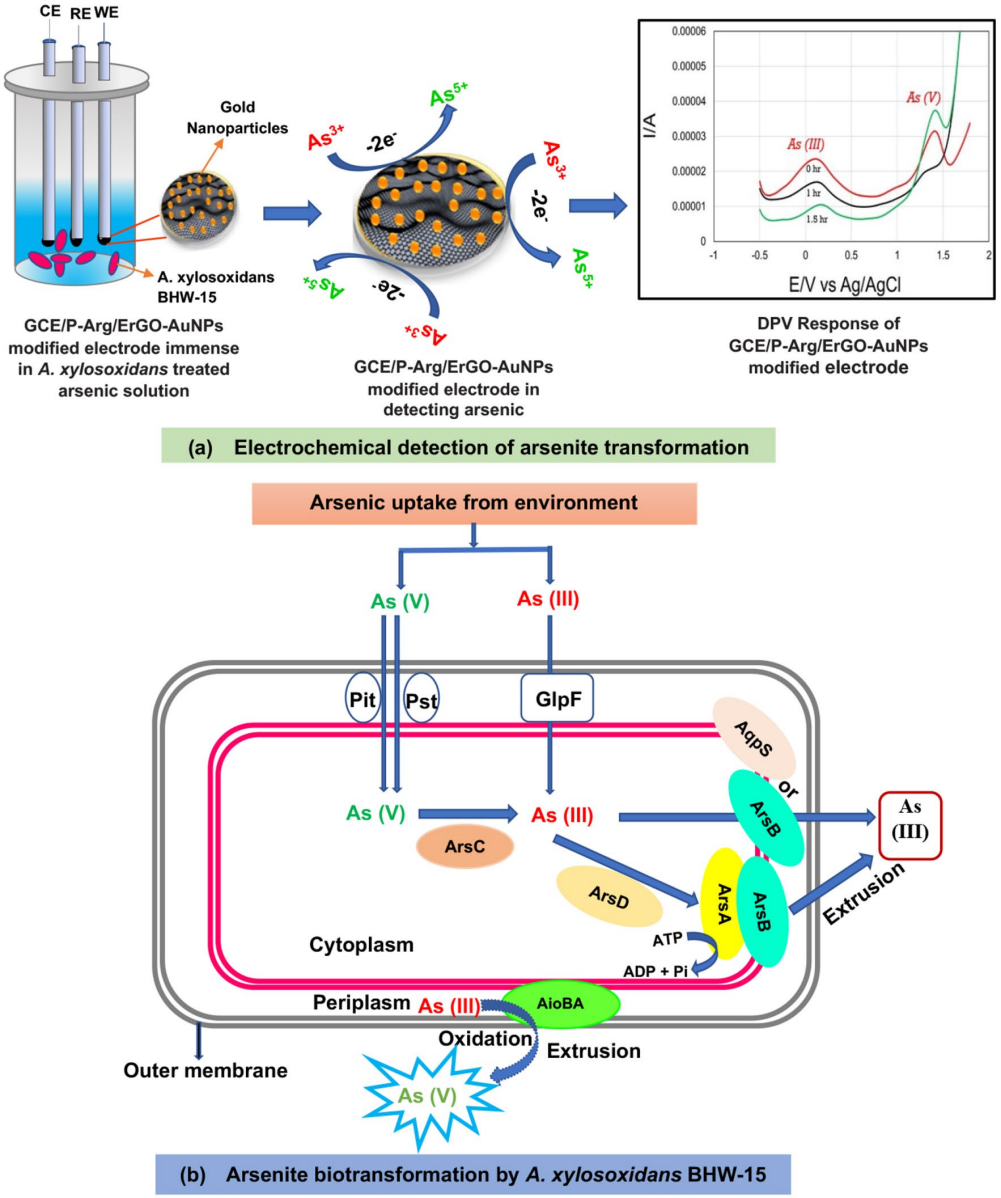
Electrochemical detection and molecular mechanism of *A. xylosoxidans* mediated As (III) biotransformation. (**a**) Electrochemical detection of arsenic transformation: GCE/P-Arg/ErGO-AuNP modified electrodes were placed in As solution treated with *A. xylosoxidans* BHW-15. In this experiment, a modified three-electrode (CE: Counter electrode, RE: Reference electrode, WE: Working electrode which was modified with gold (Au) nanoparticle) based system was used to measure the differential pulse voltammetry. The Differential Pulse voltammetry (DPV) response of arsenic on the surface of the electrode showed a remarkable peak current during the oxidation of As (III) to As (V) by the strain at a scan rate of 50 mVs^−1^. (**b**) Proposed model of As (III) biotransformation by *A. Xylosoxidans* BHW15: The bacterium uptake arsenic from the environment in different forms utilize it as an electron donor and energy source in their cell. The genome of BHW-15 carried two operons—aioSXBA (oxidation) and arsRCAD (reduction). Cytoplasmic reduction of As (V) to As (III) is occurred by the ArsC protein leading to subsequent extrusion via ArsB or ArsAB pump is pumped out from the cell by efflux pump specific ArsB transporter while, *aio *operon regulates the oxidation of the arsenite to arsenate inside the cell in presence of periplasmic arsenite oxidase (aioBA) enzyme and pumps it out form the cell using unknown efflux pump (Istiaq et al.^26^). We found the oxidation process of As (III) to As (V) by *A. xylosoxidans* BHW-15 was predominant.

To investigate the transformation mechanism, 20 µl of *Achromobacter* was added in 20 ml of 10 ppm arsenic solution. Then the mixture was kept in incubation at 25°C for a different period. Figure 6 represents the DPV of arsenic detection before and after incubation with *A*. *xylosoxidans* BHW-15. It was observed that As^3+^ started to oxidize to As^5+^ after incubation with the isolate. A complete conversion of As^3+^ to As^5+^ was noticed after 1.5 h of the incubation period. The detailed calculation of the peak current during electrochemical measurements was reported in Table 3. The electrochemical detection process of As (III) transformation is shown in Fig. 7a and a model representing the molecular interaction of *A. xylosoxidans* BHW-15 with the arsenic species is proposed based on both the experimental findings of this study and the genome sequence of *A. xylosoxidans* BHW-15^26^ (Fig. 7b).

**Table 3:** Measured peak current during the exhaustive oxidation of As^3+^ to As^5+^ by *A. xylosoxidans* BHW-15. The condition of DPV measurements was initial potential − 1.0 V, end potential 2.0 V, pulse width 50 ms, modulation 50 mV, and pulse period 0.1 s.

Incubation time (h)	Peak current for As^3+^ (µA)	Peak current for As^5+^ (µA)
0	11.35	10.22
1	3.69	23.45
1.5	0.32	14.55

## Discussion

Major health problems occurred due to the presence of high As concentration in groundwater and surrounding soil of Bangladesh^44^. Arsenite is highly toxic, soluble, and more mobile than arsenate. On the other hand, arsenate compounds are poorly soluble in water therefore, can easily be entrapped to reduce arsenic concentration in groundwater^45^. Many bacterial species have evolved various mechanisms to detoxify arsenic through oxidation, methylation, cytoplasmic, and respiratory arsenate reduction^46,47^. Using arsenite metabolizing bacteria might be an interesting approach for the development of a cost-effective and eco-friendly As detection and bioremediation model^48^. In this study, we investigated the qualitative and electrochemical As (III) transformation efficiency of a novel bacterium *Achromobacter xylosoxidans* BHW-15, and determined its potential role in As (III) bioaccumulation and immobilization.

*A*. *xylosoxidans* BHW-15 is a novel arsenite oxidizing heterotrophic beta-proteobacteria previously demonstrated by Istiaq et al.^26^. Heavy metals such as As, Cd, Pb, Cr, and others are widely known to be deadly to microorganisms. Some bacteria including arsenotrophic bacteria, however, can overcome the toxicity by developing resistance and even exploit it in their metabolic process^49^. The determination of MIC is a well-established approach that is used as a gold standard for monitoring microbial resistance in the presence of toxic chemicals such as heavy metals, antibiotics, and other contaminants. Proper microbial activity and cellular mechanisms were rarely observed to respond with a high MIC value of a toxic compound^50^. Arsenotrophic bacteria, which are mainly found in extreme habitats, use arsenic as their primary source of carbon and obtain energy for survival and metabolism. During the arsenic detoxification process, they use As compound as an electron donor and/or electron receptors^51^. In this study, we investigated our strain’s arsenic tolerance by estimating its MIC value, which proved its high tolerance and suitability for subsequent studies. Our isolate was able to resist 15 mM arsenite which surpasses all the previous reports on *Achromobacter*; for example, *Achromobacter* sp. strain N2 and *Achromobacter* KAs 3-5/N2 having MICarsenite 5 mM and *Achromobacter* *sp*. SY8 with a MICarsenite 13 mM was reported^27–29^. In this investigation, we found a correlation between the minimum inhibitory concentrations (MICs) of arsenite and the presence of energy-dependent efflux pump gene *ars*B within this isolate, as reported by Istiaq et al., Cai et al., Achour et al. and Cervantes et al.^26,52,53,54^.

SEM micrographs of BHW-15 revealed dramatic morphological and structural changes of the isolate demonstrating intracellular arsenic uptake. Numerous studies have discovered morphological alterations in bacterial shape and size as a result of heavy metals toxicity^22^. Our results depicted that the cell size and volume of BHW-15 were increased with the elevation of arsenic concentration (2 mM and 5 mM) compared to untreated cells. Many arsenic-treated cells were lysed and burst with a deep dent in their cell wall at 15 mM As concentration which was its maximum tolerance level. The actual strategy behind this was not clear and we need further investigation. However, there might be a possibility of intracellular accumulation of As at higher concentration and lower pump out of arsenic resulting in the cell burst with the death of most cells. Our findings have a good connection with the previous investigations on As uptake by microorganisms^19,55–58^. This result was also found to be consistent with the observation of Rahman et al. in case of formation of long-chain in response to arsenic stress compared to untreated cells indicating a probable tactic for accumulation of As inside their cells as well as contributing in As bioremediation^19^. Due to the combined uptake of Cd and Pb and to adapt to toxic conditions, the cell size of *Pseudomonas aeruginosa* was increased as observed by Zolgharnein^59^ which had also a similarity with our SEM imaging results. The intracellular As bioaccumulation ability make our strain useful for biological detoxification of arsenic.

Considering the bioaccumulation ability through SEM analysis, groundwater isolate *A*. *xylosoxidans* BHW-15 was immobilized in calcium alginate and the immobilized cells showed arsenite conversion up to 18 days while the substrate was changed every 6 days. After feeding the new substrate, conversion was observed on the 3rd day and the color intensity after the addition of KMnO_4_ remained the same on the 5th and 6th days. Thus, we can assume that complete conversion occurred within the 5th or 6th day. The immobilized cell converted 0.038 g sodium arsenite to arsenate within 6 days, which was more efficient than arsenite conversion using *Pseudomonas arsenicoxydans* immobilized on zeolite reported by Valenzuela et al.^60^ which can convert 0.003 g in 6 days. This investigation represented the biotechnological potential of our strain BHW-15 in arsenic detoxification.

The molybdate blue assay confirmed aerobic arsenite oxidation in our isolate. *Achromobacter xylosoxidans* BHW-15 (generation time: 4 h 15 min in the presence of arsenic) started arsenite conversion after 30 h and the conversion rate was 0.0224 mM arsenite per hour during the stationary phase of growth under aerobic conditions. It was demonstrated that strain N2 took 72 h to convert 1.0 mmol/L of arsenite to arsenate under chemoorganotrophic condition^25^. The arsenite conversion rate of 0.7211 mM/h was found in *Achromobacter* sp. SY8 was demonstrated by Lin et al.^61^. Fan et al. also reported about a potential arsenite oxidizer that started converting arsenite after 14.5 h of inoculation and converted arsenite at the rate of 0.2702 mM/h^62^.

Bacteria in the environment are exposed to a wide variety of arsenic concentrations. As a result, these bacteria evolved a metal resistance system by including the expression of resistance genes encoded in their genome in response to stress and shielding their cellular machinery from As toxicity. We previously reported the genomic structure of *A. xylosoxidans* BHW-15^26^. The genome of this strain harbored two operon- aioSXBA (oxidation) and arsRCAD (reduction). Comparative expression investigation of the arsenite oxidase (*aio*A) gene in BHW-15 revealed that the expression fold of the *aio*A gene increased to 1.6-fold under the arsenite-induced condition compared to the control condition. The presence of arsenite may have exerted selective pressure, causing the expression of arsenite oxidizing genes (aioBA) to increase. This could be due to the existence of a periplasmic protein AioX in *A. xylosoxidans* BHW-15, which binds As (III) and sends signals to AioS, causing the arsenite oxidase and other genes to be activated^26^. This result has similarities with Liu et al.^63^. *Achromobacter* *sp*. SY8 also harbored the same protein that was responsible for regulating the expression of the arsenite oxidase gene and sensing As (III)^61^. We also found a good association between the qualitative results of the arsenite oxidation and arsenite oxidase gene expression analyses in response to arsenic exposure. The extrusion of As (III) from the cell occurs is driven by two mechanisms either using an As (III)-induced ATPase encoded by ArsA or by As (III) efflux transporter encoded by ArsB. While the *aio* operon controls the conversion of arsenite to less toxic arsenate within the cell via oxidation catalyzed by the periplasmic arsenite oxidase (aioBA) enzyme, the *ars* operon precedes bacterial endogenous detoxification by reducing As (V) to As (III), and As (III) is expelled by the same efflux pathway^64^.

There are limited studies on the combined application of bacterial species with inorganic or organic nanoparticles using the electrocatalytic method for the detection of metal ions as well as their transformation mechanism^41^. Comparing with conventional analytical techniques, electrochemical detection of arsenic keen special attention due to its simple method, selectivity, sensitivity, and wide electroanalytical applications. In the current study, the electrochemical oxidation of arsenite and its subsequent transforming mechanism by reacting with *A. xylosoxidans* BHW-15 was studied using GCE/P-Arg/ErGO-AuNPs modified electrode. It was observed that this bacterium can effectively transform arsenite after a certain incubation period. The GCE/P-Arg/ErGO-AuNPs sensor can successively elucidate the electro-oxidative behavior of As (III) and As V) ions in the water matrix. This modified electrode facilitated the transfer rate of electrons and exhibited good conductivity with better sensing ability. The proposed sensing method was also owing to a good peak-to-peak separation technique and electrochemically explained the arsenite transformation ability of the isolate. Moreover, total quantification of arsenic in water samples might be possible with the help of this sensor proposed in this study.

Till to date, several arsenic-resistant bacterial species including *Achromobacter* sp. were reported which can withstand high concentrations of arsenic through the accumulation and assimilation of arsenic into biomolecules^18,65,66^, therefore, may be potential for the bioremediation or detoxification of arsenic from the arsenic-contaminated environment. However, most of them lack adequate information on their genetic makeup and quantitative efficiency. Thus, our strain has the potential to be a pioneer in this field by serving as a prospective candidate for arsenic detoxification.

## Conclusion

Our isolated *Achromobacter xylosoxidans* BHW-15 was one of the potential bacteria among other reported arsenite oxidizing *Achromobacter* sp. Besides high resistance to As (III), it also showed As accumulation and transformation at immobilized condition emphasizing its potential contribution in designing a green in situ bioremediation approach in As contaminated sites. The current study also revealed the first detailed observation of electrocatalytic As (III) transformation in *Achromobacter* sp. using a modified electrode as the electron acceptor under aerobic conditions. This research will create the scientific framework for the creation of low-cost, robust, and environmentally acceptable biosensors for the detection and measurement of arsenic using nanotechnology in future.

## Materials and methods

In this study, we selected novel arsenite oxidizing bacteria *A. xylosoxidans* BHW-15 (accession number: PZMK00000000.1) from our lab repository as a potential candidate for arsenic detection and detoxification approach. This bacterium was isolated from tube well water of Gabtali Upazila of Bogura district of Bangladesh on a hetero enriched minimal salt medium supplemented with 2 mM sodium arsenite. Therefore, we investigated the arsenic tolerance as well as bioaccumulation and immobilization capacity of this isolate. Finally, we determined the arsenite transformation potential using colorimetric and electrochemical methods.

### Arsenite tolerance assay

A heterotrophic broth medium containing different concentrations of As (III) as NaAsO_2_ (0–20 mM) was taken in each well of a 96 well plate. One row of the microtiter plate containing only medium with different As (III) concentrations was set as negative control (no inoculum added). Then the plate was incubated at 30°C for 24 h and bacterial growth was measured using Glomax Microplate Reader, USA at 600 nm.

### Scanning electron microscopy (SEM) analysis

The bioaccumulation ability of the isolate was observed using ZEISS Sigma 300 scanning electron microscopy as described by Jahid et al.^67^. Briefly, the isolate was grown in heterotrophic broth with different concentrations (2, 5, 7.5, 10, 12.5, 15 mM) of arsenite and without arsenite. 1.5 ml of cell suspension was collected into a microcentrifuge tube and centrifuged at 16,000 rpm for 5 min and the supernatant of the broth was discarded leaving the bacterial pellets. We rinsed the pellets with phosphate-buffered saline (pH 7.2) three times and fixed the cells in PBS with 4% glutaraldehyde for 2 h. Then the cells were washed three times for 15 min with PBS. Ethanol with different concentrations was used serially to treat the fixed cells for a different period (50% for 15 min, 60% for 15 min, 70% for 15 min, 80% for 15 min, 90% for 15 min, and 100% two times for 15 min each). To dehydrate the cells, they were soaked in 33, 50, 66, and 100% hexamethyldisilazane and ethanol for 15 min each. Coating of the dehydrated samples with gold–palladium was done and observed under the scanning electron microscope.

### Whole-cell immobilization of the isolate

To observe the arsenite conversion capacity of whole immobilized cells, the isolate was grown overnight in heterotrophic broth media containing 1 mM As (III). The cell pellet was taken in a sterile falcon tube and the tube was centrifuged at 4000 rpm for 20 min. The cell pellet was then added to a sterile 4% sodium alginate solution and stirred thoroughly. After that, the suspension was drawn in a sterile 5 ml syringe and added a drop at a time to 1.5% calcium chloride solution. Beads were formed and left for 10 min to harden. Then the beads were separated and added to 100 ml deionized water containing 3 mM As (III) in a conical flask. Only sodium alginate solution was also added to the calcium chloride solution by sterile syringe to form control beads that did not contain immobilized bacterial cells.

The control beads were then added to 100 ml deionized water containing 3 mM As (III) in another conical flask and incubated at 30°C. During the incubation period, arsenite transformation was tested by 0.01 M KMnO_4_ daily as described by Zahid et al.^68^. Arsenite transformation was confirmed by the presence of persistent pink color of KMnO_4_ in the supernatant showing positive phenotypic test result indicating the presence of converted arsenate. The beads were transferred to fresh deionized water containing 3 mM As (III) when there was a positive result of conversion in the previous As (III) containing water. The discoloration of pink color or formation of brownish color indicated the presence of arsenite in the supernatant that reduced the permanganate to manganese oxide showing no conversion of arsenite.

### Growth curve and arsenite conversion analysis

Bacterial growth curve analysis along with the conversion rate of arsenite was also determined. The molybdenum blue method was used to detect the arsenite transformation efficiency^69^.

Both arsenate and arsenite concentration was determined using this method^70–72^. Briefly, the isolate was inoculated in 150 ml of heterotrophic broth media containing 1 mM sodium arsenite and incubated at 30°C, 120 rpm. During incubation, periodically optical density was measured to construct a growth curve at 600 nm. Besides this, the conversion rate of arsenite to arsenate was determined. Six ml of bacterial culture was collected per hour and 2 ml culture was used to take the OD values. The remaining culture was centrifuged and the supernatant was taken for the analysis. Samples are divided into aliquots: one was kept untreated. To determine arsenate concentration, one aliquot was acidified by HCL and the acidified sample was then added with a reaction mixture containing ammonium molybdate, ascorbic acid, potassium antimonyl tartrate, and concentrated H_2_SO_4_. After that, samples were heated at 78°C for 10 min in a water bath and kept on ice for 5 min. An arsenate-molybdate complex was formed during the reaction between arsenate and molybdate which gives characteristic blue color after binding to ascorbic acid. The color intensities were measured in a spectrophotometer at 865 nm. By using a standard curve, the amount of converted arsenate at a particular stage of the growth curve was determined (Supplementary Fig. S1). For the determination of arsenite concentration, a second aliquot was oxidized in KIO_3_ and HCL and the OD_865nm_ was recorded. Arsenite concentration was measured from the difference between the oxidized and unoxidized sample.

### Analysis of arsenite oxidase (*aio*A) gene expression

The arsenite transformation ability of the isolate was also detected at a molecular level. Therefore, the bacterial arsenite oxidase gene expression was analyzed in the presence and absence of arsenic using Real-time PCR and conventional PCR methods.

### Extraction of mRNA and preparation of complementary DNA (cDNA)

For 1 ml of a culture of the arsenic-treated and untreated isolates from the log and stationary phases were collected in 1.5 ml eppendorf and centrifuged to separate the bacterial cell pellets and supernatant. The cell pellets were further treated to extract the mRNAs from the cells using PureLink™ RNA Mini Kit (Thermo Fisher Scientific, USA). Then we measured the RNA concentration through NanoDrop 2000 spectrophotometer (Thermo Scientific, USA) and the purity of prepared RNA was found good. The purified RNA was then used for cDNA synthesis. We used GoScript™ Reverse Transcription System (Promega, USA) for the preparation of complementary DNA (cDNA) and cDNA concentration was also measured.

### Primer designing of *aio*A gene for RT-qPCR

Full-length sequences of the *aio*A gene were obtained and then the sequences were compared to the GenBank database of NCBI (http://ncbi.nlm.nih.gov/GenBank) and BLAST was performed to get highly similar sequences. The sequences downloaded were analyzed using software named Molecular Evolutionary Genetics Analysis (MEGA, version 7)^73^ (https://www.megasoftware.net). A pair of primers were designed (RT *aio*A 15F: 5′-AACTCGGAATGCCATGCTAC-3′; RT *aio*A 15R: 5′-AGATTGGGGATCCAGTGATTC-3′) manually to get a conserved domain amplicon of around 150 bp for *aio*A specific RT-qPCR. Various parameters of the primers were checked by the Oligo Analyzer Tool of Integrated DNA Technologies, USA. Using the tool, Tm, GC content, length, hairpin formation, self-dimer formation, hetero-dimer formation of the primers was also verified.

### Detection of *aio*A transcript using conventional PCR

The synthesized cDNA sample of the isolate was amplified using the newly designed primer pairs and the amplicon sizes were screened by agarose gel electrophoresis to detect the expected amplicon size at around 150 bp position.

### RT-qPCR (reverse transcription-quantitative PCR) for *aio*A gene

The expression levels of the *aio*A gene at log and stationary phases of growth of the isolate in the presence and absence of arsenite was analyzed using Applied Biosystems™ 7500 Real-Time PCR System. RT-qPCR was performed in a 25 μl volume reaction mixture containing 12.5 μl GoTaq^®^ qPCR Master Mix (2×), 9.5 μl PCR grade water, 0.25 μl of each primer, 2.5 μl template cDNA and the parameters for RT- PCR cycling were as follows: 2 min hot-start activation at 95°C; denaturation at 95°C for 15 s for 40 cycles, annealing at 55°C for 1 min and dissociation at 60–95°C. SYBR^®^ was selected as the detection dye for the entire plate and a standard, two-step, 40-cycle qPCR and dissociation program was selected. The data were designated to be collected during the annealing step of each cycle. When the run was complete, the data were analyzed from the amplification plots and the curves generated by the software.

### Electrochemical sensing of arsenic with *A*.* xylosoxidans* BHW-15

#### GCE/P-Arg/ErGO-AuNPs modified electrode preparation

The modification process of the GCE/P-Arg/ErGO-AuNPs modified electrode was described in detail previously by Khan et al.^74^. Simply, we used a 0.05 μm alumina slurry to clean a bare GCE electrode to a mirror finish and it was sonicated in nitric acid (1:1), deionized water, and ethanol. After that, 2.5 mM L-Arg solution was prepared in PBS solution of pH 7.4. Later, the cyclic voltammetry (CV) method was used to electrodeposited poly L-Arg (P-Arg) onto the GCE electrode. Then, pre-synthesized gold nanoparticles (AuNPs) (15 nm) were mixed with 0.1 g/mL graphene oxide (GO) solution with sonication. After that, the GO-AuNPs composite mixture was electrodeposited on GCE/P-Arg modified electrode by CV method with a condition of − 1.2 and 0.7 V vs. Ag/AgCl and at a scan rate of 50 mVs^−1^. Finally, the electrode was named as GCE/P-Arg/ErGO-AuNPs (Supplementary Fig. S2). We purchased all the reagents for this modification from Sigma Aldrich, China. At last, ultra-pure water was used for washing the modified electrode carefully and it was stored at 4 °C for further use.

At the first stage, electro-oxidation, identification, and quantification of arsenic (As) were performed by DPV in the presence of 0.1 M phosphate buffer silane with the proposed electrochemical sensor. Later, the same arsenic solution was treated with this bacterium (about 1.4 × 10^8^ CFU/ml) and its aerobic As-transformation mechanism was studied electrochemically under room condition. A CS300 electrochemical workstation (Corrtest, Wuhan, China) was employed to conduct all electrochemical measurements. A Metrohms (DropSens) screen-printed electrode (SPE 110), three-electrode (CE, RE, WE) system was used to execute the voltammetric studies. Working and auxiliary electrodes were made of carbon, while reference electrode was available in silver or silver/silver chloride.

## Supplementary Information

Supplementary Information.
