# Supplementary material for: Bioaccumulation and detoxification of trivalent arsenic by Achromobacter xylosoxidans BHW-15 and electrochemical detection of its transformation efficiency
Source: Sci Rep. 2021 Oct 29;11:21312. doi: 10.1038/s41598-021-00745-1 (PMC8556249; doi:10.1038/s41598-021-00745-1)
Supplement: Supplementary file 1 — Supplementary Information. [file 41598_2021_745_MOESM1_ESM.docx]

**Supplementary Information**

**Supplementary Figures**


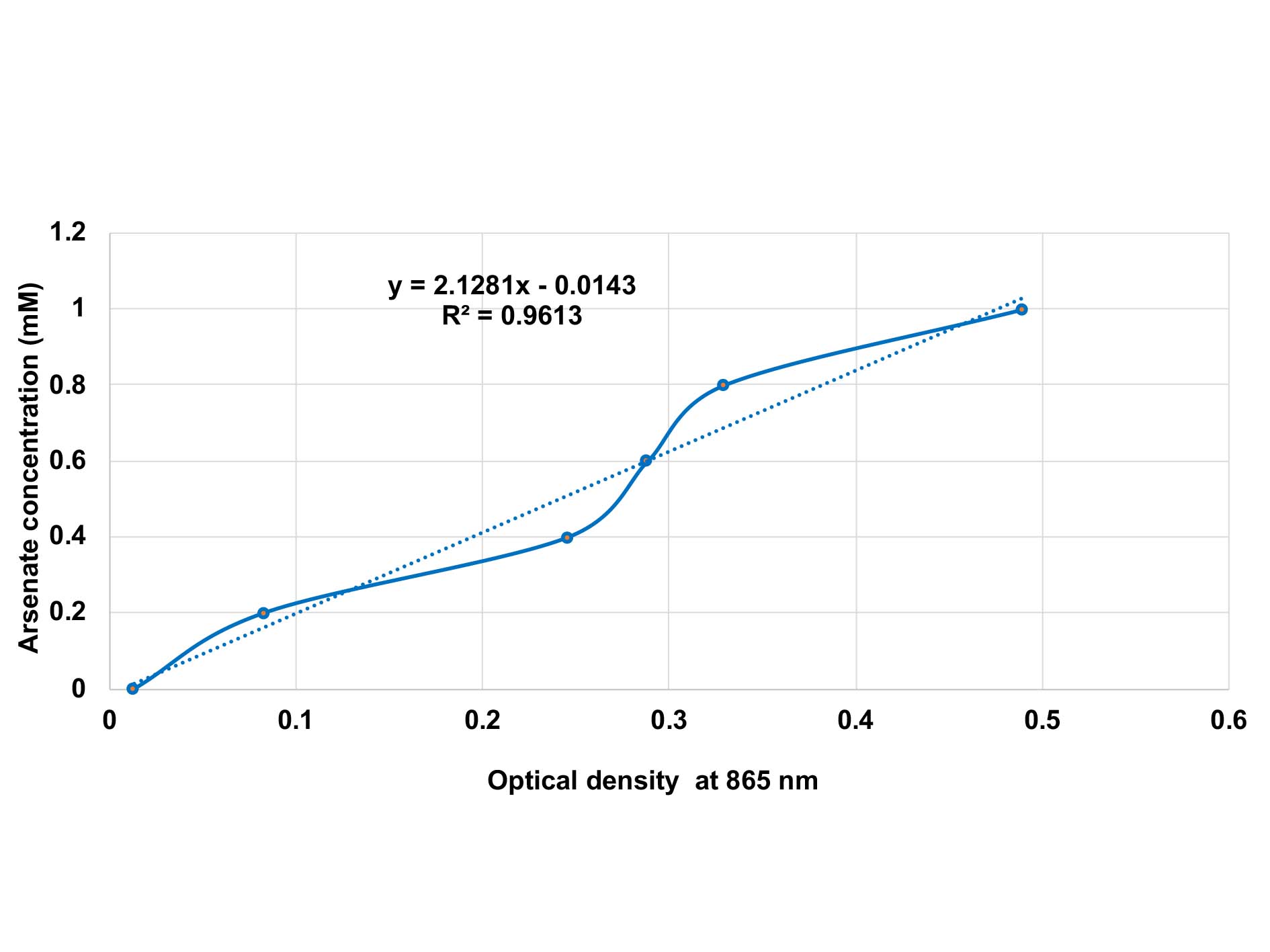


**Fig. S1** Standard curve of arsenate concentration vs absorbance at 865nm with an equation.


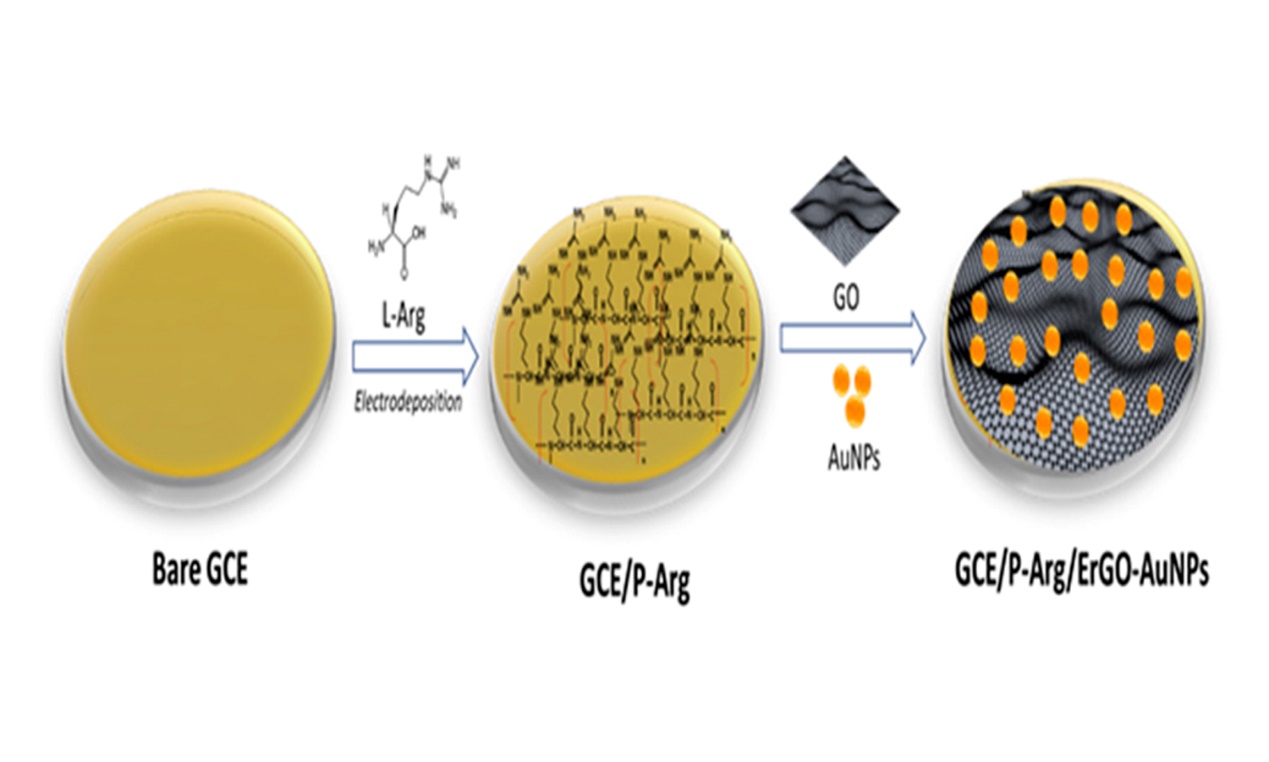


**Fig. S2** Preparation of GCE/P-Arg/ErGO-AuNPs modified electrode.


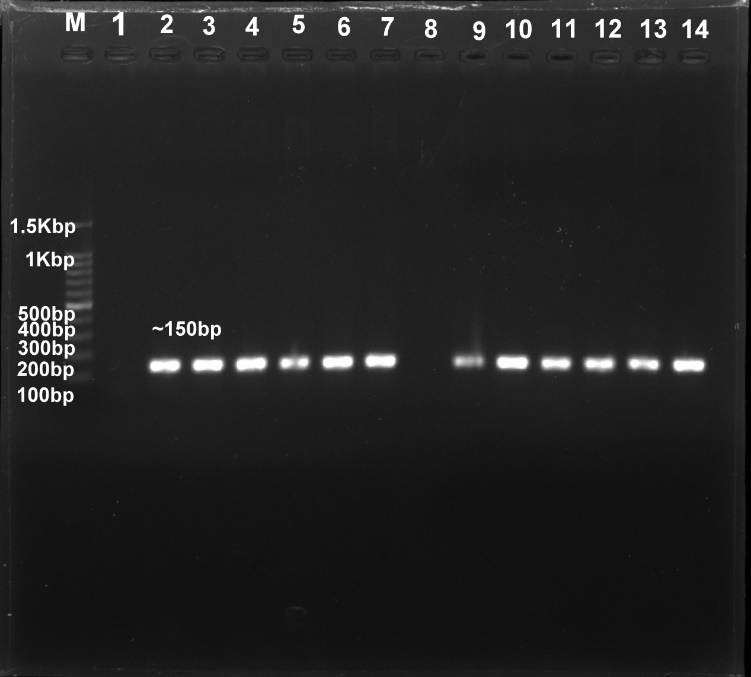


**Fig. S3:** Full-length gel of RT-*aio*A gene amplicons (~150 bp) produced from arsenic (As) untreated and treated *A. xylosoxidans* BHW-15 by conventional PCR using designed RT-primer and observed on 1% agarose gel. Here, M: Marker shown in base pairs; 1: Negative control; 2: As untreated BHW-15; 3: As treated BHW-15; 4-14 (not related to this manuscript): RT-*aio*A gene amplicons screened from other arsenite oxidizing bacteria of our lab repository.


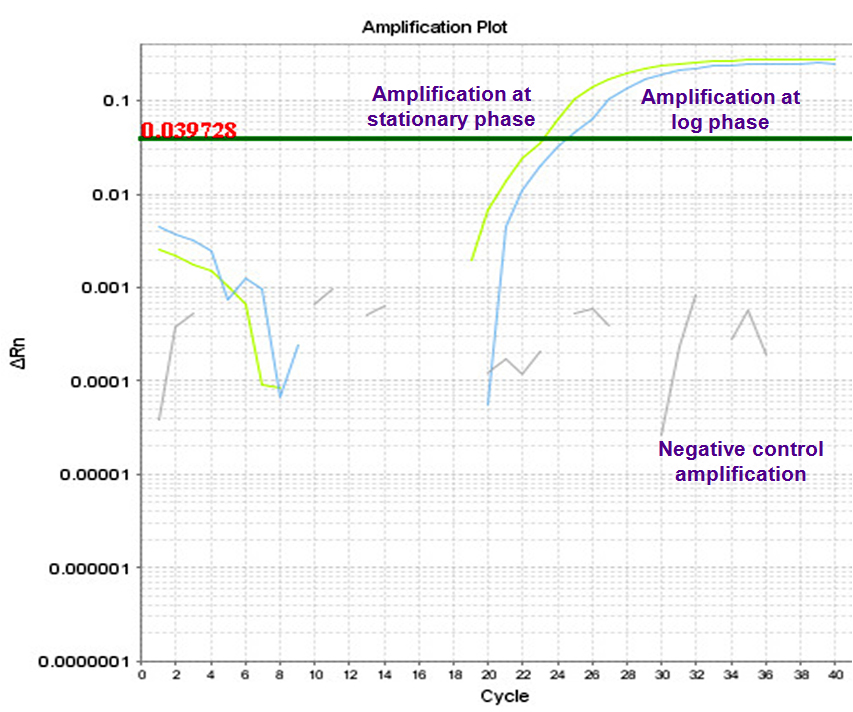


**a**


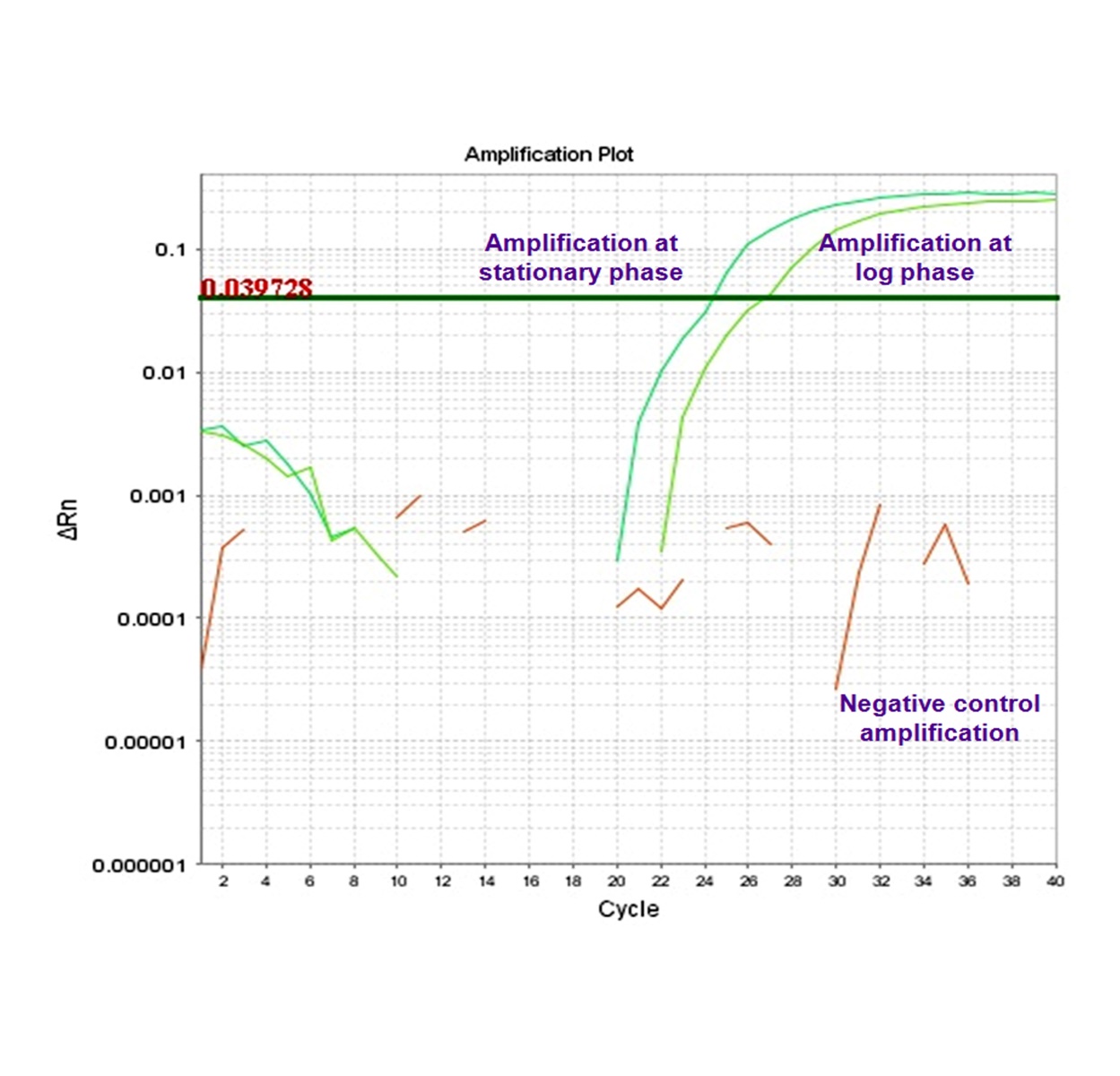


**b**

**Fig. S4** Representative amplification plot of BHW-15 for (a) only media control; (b) arsenite media during RT-qPCR.
